# Ultraviolet disinfection of *Schistosoma mansoni* cercariae in water

**DOI:** 10.1371/journal.pntd.0009572

**Published:** 2021-07-06

**Authors:** Lucinda Hazell, Fiona Allan, Aidan M. Emery, Michael R. Templeton

**Affiliations:** 1 Department of Civil and Environmental Engineering, Imperial College London, London, United Kingdom; 2 Wolfson Wellcome Biomedical Laboratories, Department of Life Sciences, Natural History Museum, London, United Kingdom; North Carolina State University, UNITED STATES

## Abstract

**Background:**

Schistosomiasis is a parasitic disease that is transmitted by skin contact with waterborne schistosome cercariae. Mass drug administration with praziquantel is an effective control method, but it cannot prevent reinfection if contact with cercariae infested water continues. Providing safe water for contact activities such as laundry and bathing can help to reduce transmission. In this study we examine the direct effect of UV light on *Schistosoma mansoni* cercariae using ultraviolet light-emitting diodes (UV LEDs) and a low-pressure (LP) mercury arc discharge lamp.

**Methodology:**

*S*. *mansoni* cercariae were exposed to UV light at four peak wavelengths: 255 nm, 265 nm, 285 nm (UV LEDs), and 253.7 nm (LP lamp) using bench scale collimated beam apparatus. The UV fluence ranged from 0–300 mJ/cm^2^ at each wavelength. Cercariae were studied under a stereo-microscope at 0, 60, and 180 minutes post-exposure and the viability of cercariae was determined by assessing their motility and morphology.

**Conclusion:**

Very high UV fluences were required to kill *S*. *mansoni* cercariae, when compared to most other waterborne pathogens. At 265 nm a fluence of 247 mJ/cm^2^ (95% confidence interval (CI): 234–261 mJ/cm^2^) was required to achieve a 1-log_10_ reduction at 0 minutes post-exposure. Cercariae were visibly damaged at lower fluences, and the log reduction increased with time post-exposure at all wavelengths. Fluences of 127 mJ/cm^2^ (95% CI: 111–146 mJ/cm^2^) and 99 mJ/cm^2^ (95% CI: 85–113 mJ/cm^2^) were required to achieve a 1-log_10_ reduction at 60 and 180 minutes post-exposure at 265 nm. At 0 minutes post-exposure 285 nm was slightly less effective, but there was no statistical difference between 265 nm and 285 nm after 60 minutes. The least effective wavelengths were 255 nm and 253.7 nm. Due to the high fluences required, UV disinfection is unlikely to be an energy- or cost-efficient water treatment method against schistosome cercariae when compared to other methods such as chlorination, unless it can be demonstrated that UV-damaged cercariae are non-infective using alternative assay methods or there are improvements in UV LED technology.

## Author summary

Schistosomiasis is a waterborne disease that affects approximately 200 million people globally. It is transmitted through contact with freshwater that contains parasitic larvae known as cercariae and is prevalent in communities that do not have access to safe water supplies. Treatment with the drug praziquantel is effective against adult worms, but this cannot prevent reinfection if contact with contaminated water continues. Providing safe water for laundry and bathing can prevent transmission of the disease and the emerging technology of ultraviolet light-emitting diodes could offer a chemical free option for point-of-use water treatment, but there is insufficient published data to determine the UV fluence-response of schistosome cercariae. In this study we investigate the effectiveness of UV disinfection at four wavelengths against *Schistosoma mansoni* cercariae. The results show that high UV fluences are required to kill *S*. *mansoni* cercariae at all wavelengths, but they are visibly damaged at lower fluences. This suggests UV disinfection alone is unlikely to be an efficient method for providing cercariae-free water unless there are improvements in UV-LED technology or further research can demonstrate UV-damaged cercariae are non-infective.

## Introduction

Schistosomiasis is a waterborne neglected tropical disease (NTD) caused by parasitic worms of the genus *Schistosoma*. NTDs are a group of diverse communicable diseases that mainly affect the poorest communities in tropical and sub-tropical regions, particularly where there is insufficient access to safe water, sanitation, and hygiene (WASH). According to the latest Global Burden of Disease study, 190 million people are currently infected with schistosomes worldwide, whilst the Global Health Observatory data states the total number of people requiring preventative chemotherapy with praziquantel was almost 230 million people in 2018 [1,2].

Infection occurs by skin contact with water containing larvae, known as cercariae, which are produced by asexual reproduction within freshwater intermediate snail hosts. Schistosome cercariae penetrate human skin and, once inside the body, develop into worms that live in the blood vessels of the urogenital or intestinal systems, depending on the species. Adult worm pairs produce eggs that are released in the urine or feces. Where there is no access to sanitation, eggs may be released into waterbodies, allowing for the infection of intermediate host snails and continuation of the lifecycle [3]. The drug praziquantel is an effective treatment, however it does not prevent reinfection if contact with cercariae infested water continues.

Access to clean water for contact activities (e.g. bathing and laundry) is a key intervention for preventing schistosomiasis in the World Health Organization’s (WHO) toolkit for WASH and NTD programs [4], and studies have shown a direct link between access to clean water and a reduction in schistosomiasis infection [5–10]. However, a systematic review found there was insufficient data to develop guidelines for treating water against schistosome cercariae, making it difficult to implement WASH programs that directly target schistosomiasis transmission [11].

Large-scale UV disinfection of water and wastewater has been used in parts of Europe since the 1950’s, and more widely in North America and Asia since the 1990’s [12]. It has the benefits of forming no regulated by-products (such as trihalomethanes or haloacetic acids produced by chlorination) and it is highly effective against chlorine-resistant pathogens such as *Cryptosporidium parvum* and *Giardia lamblia* [13,14]. UV light can be categorized into UV-A (400–315 nm), UV-B (315–280 nm), UV-C (280–200 nm), and Vacuum UV (200–100 nm), and 200–300 nm is often referred to as the germicidal range, as this is most efficient for disinfection. Conventional UV technology utilizes mercury arc discharge lamps. These require specialist handling due to the fragile quartz sleeve and mercury contents, which add risk of technology failure and health consequences to small scale, point-of-use water treatment systems. In contrast, UV LEDs are well suited to these applications. They are mercury-free, durable, and small, so they can be fitted into battery- or solar-powered pumps or water bottles [15]. A recent study evaluated a “zero-energy” UV LED reactor, which used a dynamo to convert the mechanical energy generated by hand pumping water to electrical energy for operating the LEDs [16]. The optical power of UV LEDs is currently relatively low compared to mercury arc lamps, meaning longer exposure times are required to achieve sufficient inactivation of pathogens. The wall plug efficiency (WPE, ratio of optical output power to electrical input power) of mercury arc lamps is 30–40% [17], compared to approximately 10% for the best available UV LEDs in the germicidal range [18,19]. However, this is an increase from ~1% in 2015, with predictions suggesting that the WPE of UV LEDs at 260–280 nm could exceed 20% by 2025, assuming they follow the trend of blue LEDs [19,20]. Furthermore, the cost of germicidal UV LEDs has decreased from over 1000 USD/mW in 2003 to less than 1 USD/mW today [21], with predictions they will be commercially available for less than 1 USD/W by 2030 [19].

The biological effectiveness of UV disinfection is primarily attributed to a photochemical reaction in the nucleic acids when they absorb UV light. This results in a new bond, known as a dimer, between two adjacent pyrimidine bases (primarily thymine in DNA) [22]. Dimers disrupt the structure of DNA and RNA, if a critical number are formed they prevent replication and transcription. Low pressure (LP) mercury arc lamps are the most commonly used UV sources for water disinfection as they emit near monochromatic light at 253.7 nm, which is close to the maximum absorption of the pyrimidine bases at 260–265 nm. Studies with medium pressure (MP) mercury arc lamps, which emit polychromatic light, and UV LEDs which are also polychromatic but can be tuned to various peak wavelengths, have investigated other disinfection mechanisms across the germicidal range such as protein damage and oxidative stress [23–26].

UV dose, or fluence, is measured in mJ/cm^2^ and is a product of exposure time (s) and fluence rate (mW/cm^2^), which is a function of the optical power output of the UV source. Our recent systematic review estimated that a fluence of 5–14 mJ/cm^2^ at 253.7 nm was required to prevent schistosome cercariae from developing into adult worms in animal hosts and achieve a 1-log_10_ reduction in worm burden (90% reduction) [27–36]. However, the majority of the studies in the review were carried out before the now widely accepted standard protocol for determining UV fluence was published by Bolton and Linden in 2003 [37]. It is unclear if the measurements accounted for the distribution of UV light over the sample surface or the reflection, absorption, or divergence of light as it travelled through the sample; the fluences recorded should therefore be considered approximate at best. Furthermore, these results were from vaccine studies and did not quantify the direct effect on the cercariae. At these fluences cercariae were visibly unharmed and were still able to actively find and infect a host (penetrate the skin), but they were not able to develop into adult worms.

Using infectivity studies to determine the effectiveness of water treatment processes against schistosome cercariae requires animal testing and was therefore not considered for this study. In addition, these studies may not provide the most reliable fluence-response curves for helminths, as worm recovery may not be directly proportional to the number of organisms in the inoculant [36,38]. In a study on *Echinococcus granulosus* 75% of mice developed infections if they were inoculated with 2,000 eggs, but this reduced to 0% when the number of eggs was 500 [39]. Another study on *Taenia taeniaeformis* found that the number of worms recovered from control animals varied from 4–30% of the number of eggs in the inoculant [40]. This was thought to be because of incomplete recovery of worms or because of an unknown health condition which caused a reduction or increase in infection intensity in some of the animal hosts.

Skin penetration by schistosome cercariae can cause cercarial dermatitis and migration of the parasite around the body before it fully develops into an adult worm may result in Katayama syndrome in sufferers of acute schistosomiasis. Symptoms of Katayama syndrome include sudden fever, headaches, muscle pain, abdominal tenderness, malaise, and fatigue [41,42]. Therefore, to make water safe from schistosome cercariae, treatment processes should aim to prevent skin penetration entirely.

Some previous studies have considered the direct effect of UV light on schistosome cercariae, demonstrating that short exposures to UV light reduced motility and skin penetration [43–45], but the fluence rate was not recorded in any of these studies and the experiments cannot be reproduced. In this study we examine the direct effect of UV disinfection on *Schistosoma mansoni* cercariae by studying the morphology and motility of cercariae post-UV exposure to determine if they have been killed. Tests were conducted with a LP lamp and UV LEDs at three peak wavelengths: 255 nm, 265 nm, and 285 nm, using previously published protocols for fluence determination [37,46].

## Method

### Ethics statement

The complete life cycle of *S*. *mansoni* NMRI (Puerto Rican) strain is maintained at the Wellcome Sanger Institute (UK). All the regulated procedures were conducted under the Home Office Project License No. P77E8A062, and protocols were revised and approved by the Animal Welfare and Ethical Review Body (AWERB) of the Wellcome Sanger Institute. The AWERB is constituted as required by the UK Animals (Scientific Procedures) Act 1986 Amendment Regulations 2012.

### Intermediate host snail infection

Snail infections were carried out at the Natural History Museum (London, UK) with *S*. *mansoni* from infected mouse livers obtained monthly from the Wellcome Sanger Institute. Eggs were extracted from the livers using a Pitchford funnel and hatched into miracidia in bottled spring water (Volvic, pH 7, 27°C). To ensure even exposure, thirty to fifty *Biomphalaria glabrata* snails were individually placed into separate beakers, each containing 20 mL of bottled water and 5 miracidia, for 12 hours. Snails were transferred to the Roger Perry Laboratory at Imperial College London (UK) in the prepatent period and maintained at 27°C. Cercarial production commenced 25–30 days post-infection. Snails were kept in the dark for 24 hours prior to all experiments, to allow for maximal shedding of cercariae.

### Preparation of cercariae and enumeration

Five to ten infected snails were selected at random and rinsed in bottled water. Snails were transferred to a beaker containing 5–8 mL of bottled spring water (Volvic, pH 7, 27°C) and placed under bright, visible light for 60–90 minutes, to induce shedding of cercariae. After this time, the snails were removed and the cercariae infested water was filtered through a 200 μm polyester mesh to remove snail feces. Three 100 μL aliquots were taken by pipette and 10 μL of Lugol’s iodine was added to fix and stain the cercariae so they could be easily counted. Based on the mean concentration, the volume of infested water required to achieve 100–120 cercariae per sample was calculated (typically 200 μL of cercariae infested water per sample, maximum 400 μL). Fresh cercariae were shed daily for each set of UV exposures and all exposures were completed within a maximum of six hours of the cercariae having been shed from a snail.

### Irradiance measurement and fluence determination

**Fig 1:**
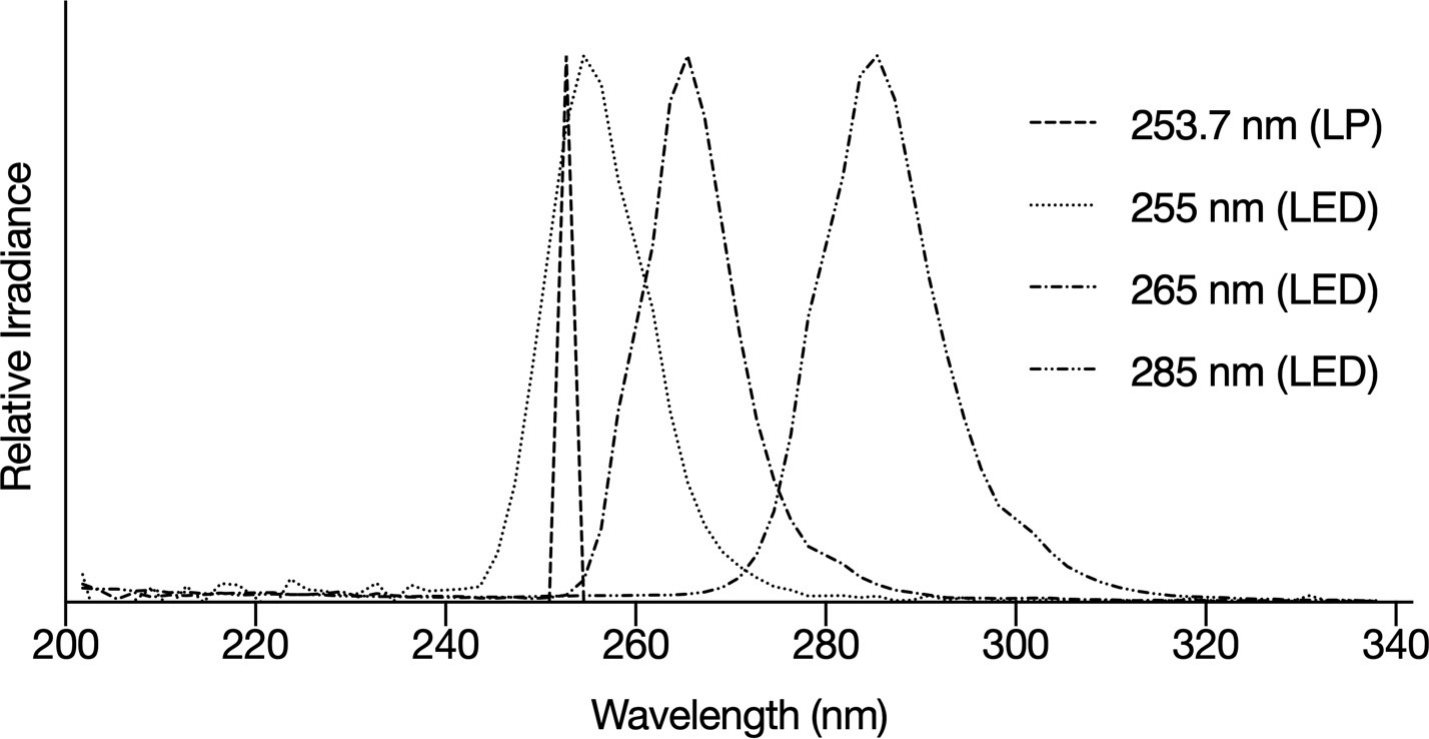
Emission spectra of the UV LEDs and LP lamp.

Cercarial suspensions were exposed to polychromatic UV light at three peak wavelengths, 255 nm, 265 nm, and 285 nm, using a Triple Wavelength PearlLab Beam UV LED Device (Aquisense Technologies, Kentucky, USA). The device is equipped with three LEDs at each wavelength (which can be operated independently or simultaneously), power control, thermal management, and an optimizing tube. The technical specifications of the LEDs are shown in Table 1. For comparison, cercariae were also exposed to near monochromatic radiation from a LP lamp, emitting at 253.7 nm, set up as a collimated beam. The emission spectra of the UV LEDs and the LP lamp are shown in Fig 1.

**Table 1:** Technical specifications of UV LEDs.

	255 nm	265 nm	285 nm
**Peak wavelength (nm)**	255.4	265.1	284.1
**Weighted average wavelength (nm)**	257.4	266.3	284.3
**Full width half maximum (nm)**	14.3	10.9	13.3

The incident irradiance at the sample surface was measured using an ILT 1700 radiometer equipped with a SED 270 detector (both International Light Technologies, calibrated annually). All UV LED sources were switched on at least 15 minutes before measuring the irradiance to allow the outputs to stabilize, the LP lamp was switched on 30 minutes before measurement. UV LED irradiance measurements were recorded at 5 mm increments across the entire sample surface and the weighted average incident fluence rate and corresponding coefficient of variation (CV) were calculated [46]. A sensor factor was applied to account for the polychromatic output of the UV LEDs. The LP irradiance measurements were recorded at 5 mm increments along the orthogonal axis of the sample surface. The Petri factor was calculated and applied to the incident irradiance measurement at the center of the sample surface [37]. For the LEDs a CV less than 6.7% indicates a uniform irradiance distribution over the surface of the sample and is equivalent to a Petri factor greater than 0.9 for a LP collimated beam [46]. CV values ranged from 2.0% to 2.4% for all UV LED exposures, and Petri factors from 0.9813 to 0.9942 for all LP exposures. Irradiance measurements were repeated following the completion of all UV exposures, to confirm they were within 5% of the initial measurement [47].

To determine the average fluence rate in the sample, factors were applied to incident irradiance measurements to correct for the absorbance (water factor), divergence, and reflection of UV light [37]. To calculate the water factor, a UV-2401PC spectrophotometer (Shimadzu, UK) was used to measure the absorbance of the sample at the weighted average wavelength of the UV LEDs and the peak wavelength of the LP lamp. The absorbance was less than 0.02 cm^-1^ for all experiments, therefore the water factor was not weighted to the spectral power distribution for the polychromatic UV LEDs [46].

Exposure times (s) were calculated by multiplying the required fluence (mJ/cm^2^) by the inverse of the average fluence rate (mW/cm^2^) in the sample. The average fluence rates were 0.053 mW/cm^2^, 0.199 mW/cm^2^, and 0.6923 mW/cm^2^ for the 255 nm, 265 nm, 285 nm UV LEDs respectively and 0.214 mW/cm^2^ for the 253.7 nm LP lamp.

### UV exposure procedure

Stirred suspensions of 3.8 mL (0.6 cm deep, 3 cm diameter) containing approximately 100–120 cercariae were exposed at distance of 10.4 cm from the LED source and 31.5 cm from the LP source for the required time. Samples were exposed in a random order to a UV fluence of either 0, 50, 100, 150, 200, or 300 mJ/cm^2^ (in duplicate or triplicate), to generate a UV fluence-response curve for each wavelength.

A manual shutter was used to control the exposure times which varied from 1 minute 11 seconds to 95 minutes 19 seconds, depending on the fluence and wavelength of light being used. As the optical power output of the UV LEDs decreases with wavelength, the exposure times for the 255 nm LEDs were much longer than for the 285 nm LEDs (e.g. 31 minutes 38 seconds and 2 minutes 27 seconds to achieve a fluence of 100 mJ/cm^2^ using 255 nm and 285 nm LEDs, respectively). The procedure for control samples (0 mJ/cm^2^) was the same as for test samples, except the shutter remained closed. Control samples were “exposed” for 10 minutes. All experiments were conducted at least twice, using cercariae shed from different snail batches.

### Enumeration of cercariae post-exposure

Samples were examined beneath a stereo-microscope after UV exposure and the motility and morphology of cercariae on the bottom of the Petri dish were studied to determine if they were alive or dead. A dissecting needle was used to gently nudge cercariae to see if they would move. Non-moving cercariae that had turned opaque with everted suckers and had fully relaxed tails, often separated from the head, were considered dead. Cercariae that were moving or had contracted, rigid tails with tightly curled forks were considered alive.

Initially samples were studied immediately following exposure to UV light, however it became clear that UV-damaged cercariae may not die immediately, even when exposed to high fluences. The experiments were repeated, and samples were studied at 0, 60, and 180 minutes (± 3 minutes) post-exposure. Data from initial experiments were analyzed with data at 0 minutes post-exposure from subsequent experiments. All samples were kept in the dark between counting at 60 and 180 minutes post-exposure. Following final enumeration of dead cercariae, 20 μL of Lugol’s iodine was added to the Petri dish to fix and stain all cercariae and the total was counted.

Cercariae sink if they are not actively swimming, therefore only cercariae that were on the bottom of the Petri dish were counted and where heads and tails had separated only heads were counted to avoid double counting. Surface tension would occasionally trap dead cercariae at the surface of the sample, although this was rare and when it did occur it would only affect one or two cercariae. Cercariae on the sample surface were not included when counting the dead or total number of cercariae.

### Statistical analysis

At least four replicate samples were assayed at each fluence and the total number of cercariae per sample ranged from 96 to 143 (mean 112). The number of alive cercariae was calculated by taking the number of dead from the total number of cercariae in each sample and the log_10_ reduction was calculated as *log*_*10*_ *(N*_*0*_*/N)*, where *N*_*0*_ is the mean proportion of alive cercariae in the control (0 mJ/cm^2^) samples and *N* is the proportion of alive cercariae in the experimental samples at the same time point (i.e. 0, 60, or 180 minutes post-exposure). For the log_10_ reduction of control samples at 60 and 180 minutes post-exposure *N*_*0*_ is the proportion of alive cercariae in the control samples at 0 minutes post-exposure. In samples where there were no surviving cercariae the minimum log_10_ reduction has been determined by assuming one cercaria had survived.

Fluence-response data in the linear region were fit using least squares regression in GraphPad (Prism version 9.0.2) and the log_10_ fluence-based inactivation rate constant was determined as follows:

$${log}_{10}\frac{N_{0}}{N}=k_{D}\times F_{\lambda }+c$$

Where *k*_*D*_ is the inactivation rate constant, and *F* is the fluence at wavelength λ. All data sets exhibited a shoulder at low fluences therefore the fluence-response data was fit with a constant term *c*, representing the y-intercept. Samples that had no surviving cercariae were excluded from the regression along with data points that were in the shoulder region of the fluence response curve (determined visually).

Inactivation rate constants were compared using a one-way ANOVA (α = 0.05) followed by Tukey’s multiple comparisons test. If the inactivation rate constants of two wavelengths were statistically similar (*p* > 0.05), an extra-sum-of-squares *F-*test (α = 0.05) was carried out to determine if the two data sets could be fit to a shared model, to confirm similarity.

To assess the impact of stirring at long exposure times a test was also carried out to compare samples kept in the dark for 10, 30, and 90 minutes; half of the samples were stirred and half were unstirred. Dead cercariae were counted at 0, 60, and 180 minutes after stirring, and all samples were kept in the dark (unstirred) between counts. The proportion of alive cercariae in stirred and unstirred samples was compared using an unpaired two-tailed *t-*test (α = 0.05).

## Results

### Visible damage caused by UV exposure

**Fig 2:**
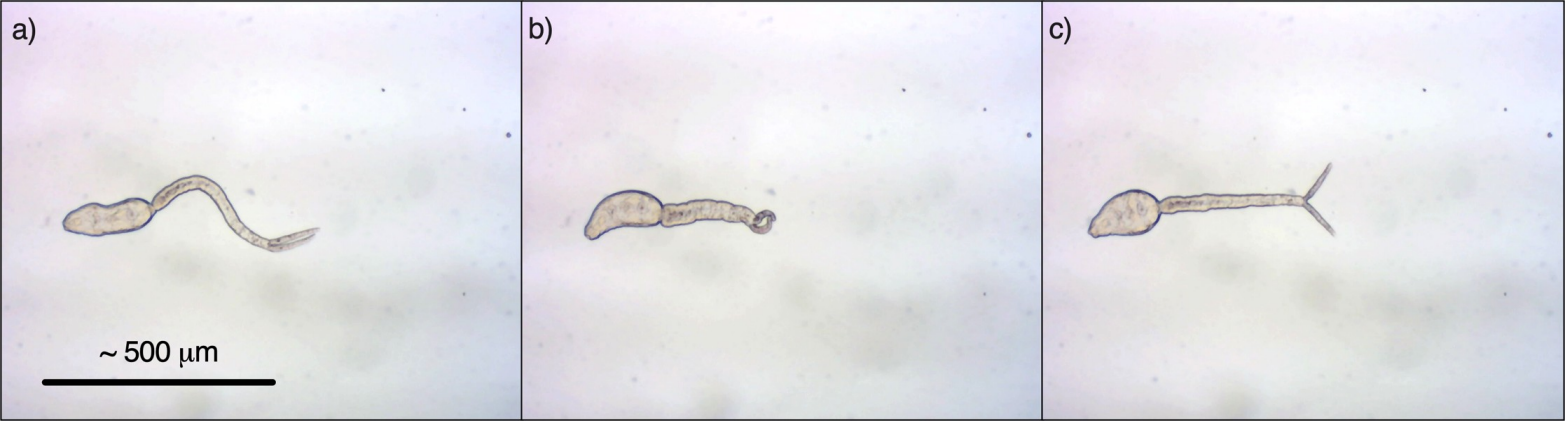
Series of photos showing tail thrusting and stretching and contraction of a *S*. *mansoni* cercaria from a control sample (0 mJ/cm^2^), 40× magnification.

Non-UV exposed cercariae in control samples were observed actively swimming near the sample surface with their tails thrusting quickly from side to side, giving the appearance of spinning (Fig 2). When kept in the dark between counting, cercariae became sedate and sunk to the bottom of the Petri dish, however when exposed to bright visible light under the microscope they were quickly stimulated and swam back to the surface. Only cercariae heads that had separated from the tails would remain on the bottom of the Petri dish, however they were few in number and mostly still alive. It is thought that these heads became separated either during pipetting or because of stirring.

**Fig 3:**
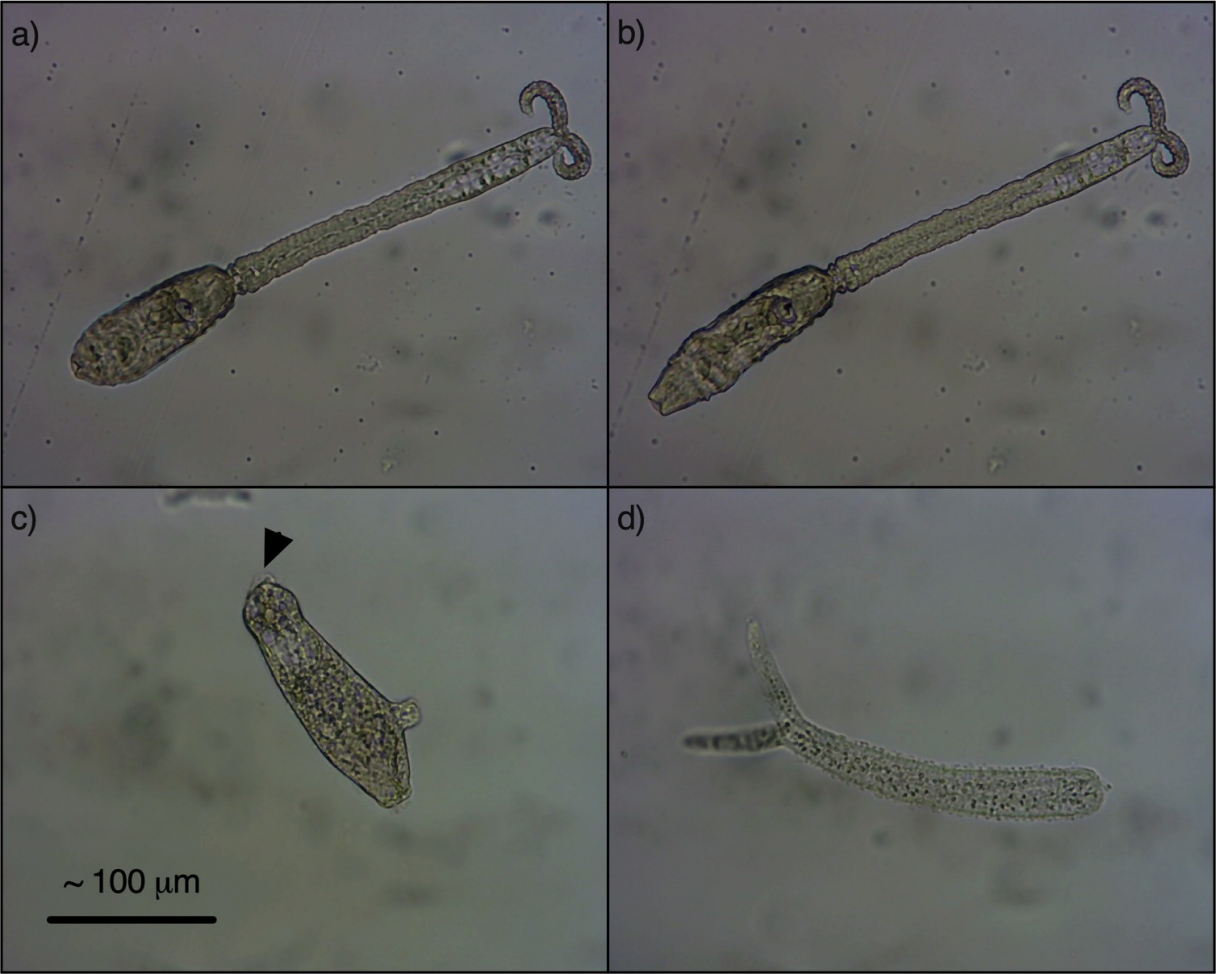
A and B) Alive but damaged *S*. *mansoni* cercaria exposed to 100 mJ/cm^2^ at 265 nm showing rigid tail with tightly curled fork and contraction and stretching of head. C and D) Dead *S*. *mansoni* cercaria exposed to 300 mJ/cm^2^ at 265 nm, showing fully everted oral and ventral sucker with gland contents just visible (arrow) and fully relaxed, detached tail. All 100× magnification.

Following exposure to increasing fluence of UV light at all wavelengths, cercariae became lethargic and swimming slowed. Periods of non-swimming exceeded periods of swimming, and cercariae began to sink, eventually reaching the bottom of the Petri dish. Here they attempted to swim, but movements were slow or in short burst of twitching, and they were unable to swim back to the surface. Some tails detached but this did not necessarily indicate death and cercariae heads would continue to contract and stretch. Where tails remained attached, they often appeared rigid, with tightly curled forks (Fig 3A and 3B).

At higher fluences, and as time post-exposure increased, movement slowed further and became even more intermittent as the oral and ventral suckers began to invert and evert. Cercariae became sticky, possibly because of gland contents being expelled from the suckers or released at the head-tail junction when detachment occurred. Dead cercariae had opaque, swollen heads with fully everted suckers (Fig 3C). This changed the shape of the head, so it appeared slightly triangular at low magnification. The tails became fully relaxed (Fig 3D), floating slightly above the head, and easily detached if the sample was disturbed or the cercariae were nudged with a needle. Morphological changes were the same at all wavelengths, but they did not necessarily occur at the same fluence or time post-exposure. Cercariae exposed to 50 mJ/cm^2^ did not appear to be immediately damaged at 253.7 nm or 255 nm, however many alive cercariae had detached tails 60 minutes post-exposure. At 265 nm and 285 nm the damage caused by 50 mJ/cm^2^ was more immediate, although a small number of cercariae were still actively swimming at 60 minutes post-exposure. The majority of cercariae were visibly damaged immediately following exposure to 100 mJ/cm^2^ at all wavelengths, however after 3 hours many were still alive in the 253.7 nm and 255 nm samples.

### Fluence-response

**Fig 4:**
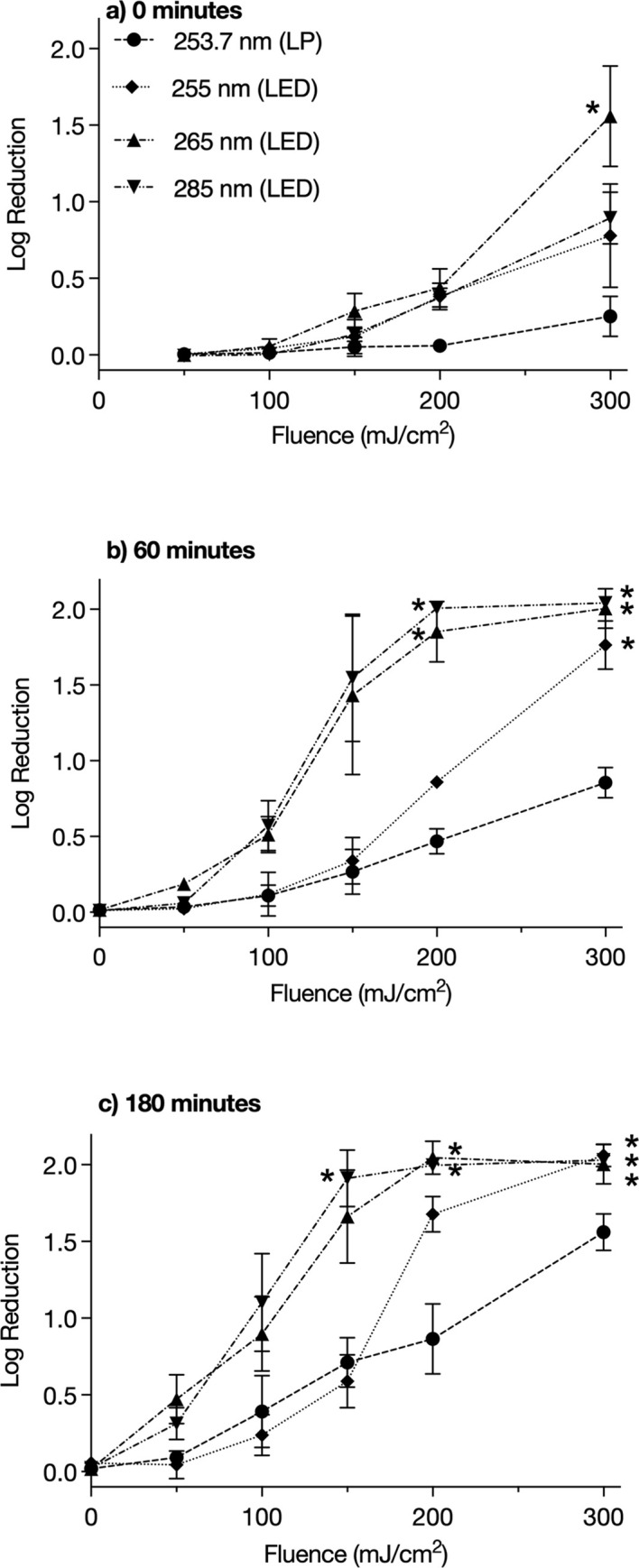
UV fluence-response of *S*. *mansoni* cercariae at A) 0 minutes, B) 60 minutes, and C) 180 minutes after exposure to UV light at four wavlengths. * indicates all cercariae were dead in at least one sample and therefore represents the minimum log_10_ reduction achieved. Error bars indicate ±1 standard deviation.

The mean fluence-response of *S*. *mansoni* cercariae to UV exposure at four wavelengths using UV LEDs and a LP lamp at 0, 60, and 180 minutes post-exposure is shown in Fig 4, with error bars representing one standard deviation. All data points for 0, 60, and 180 minutes post-exposure are shown in S1–S3 Figs, respectively. The inactivation rate constants and interpolated fluences required to achieve a 1- and 2-log_10_ reduction at each wavelength and time point are shown in Table 2 (full inactivation equations are shown in S1–S3 Figs).

**Table 2:** Inactivation rate constants (*k*_*D*_) of *S*. *mansoni* cercariae for each wavelength at 0, 60, and 180 minutes post-exposure and the fluence required to achieve 1- and 2- log_10_ reduction.

			Fluence (mJ/cm^2^) (Range for 95% CI)	
**0 mins**	***k***_***D***_	**±Std. Err**	**1-log**_**10**_	**2-log**_**10**_
253.7 nm (LP)	0.0012	0.00017	> 300	> 300
255 nm (LED)	0.0038	0.00039	> 300	> 300
265 nm (LED)	0.0073	0.00050	247 (234–261)	> 300
285 nm (LED)	0.0046	0.00021	> 300	> 300
**60 mins**	***k***_***D***_	**±Std. Err**	**1-log**_**10**_	**2-log**_**10**_
253.7 nm (LP)	0.0038	0.00033	> 300	> 300
255 nm (LED)	0.0081	0.00048	218 (209–229)	> 300
265 nm (LED)	0.0113	0.00162	127 (111–146)	216 (188–266)
285 nm (LED)	0.0130	0.00141	126 (115–141)	202 (180–239)
**180 mins**	***k***_***D***_	**±Std. Err**	**1-log**_**10**_	**2-log**_**10**_
253.7 nm (LP)	0.0057	0.00043	208 (194–225)	> 300
255 nm (LED)	0.0105	0.00124	160 (145–179)	255 (226–302)
265 nm (LED)	0.0119	0.00174	99 (85–113)	183 (160–226)
285 nm (LED)	0.0155	0.00163	94 (84–104)	158 (143–181)

All wavelengths demonstrated a shoulder at low fluences when studied immediately after exposure (0 minutes). Whilst cercariae were visibly damaged at all wavelengths, the mean maximum log_10_ reduction achieved immediately following exposure to the highest fluence was 1.6 at 265 nm. In only one sample did all the cercariae die, when exposed to 300 mJ/cm^2^ at 265 nm. The inactivation rate constants at 0 minutes post-exposure were statistically different for all UV sources (*p* < 0.0001), except for 255 nm vs. 285 nm (*p* = 0.45). A subsequent extra-sum-of-squares *F*-test confirmed similarity of the curves (*p* = 0.23). Refer to S1 Table for *p* values for all multiway comparisons.

Sixty minutes after exposure there was an increase in log_10_ reduction at all fluences and wavelengths, with the most notable increase at 285 nm. All wavelengths still exhibited a shoulder up to 50 mJ/cm^2^. All cercariae were dead in all samples at 300 mJ/cm^2^ at 265 nm and 285 nm and in one sample at 255 nm, and in all samples at 200 mJ/cm^2^ at 285 nm and in half of the samples at 265 nm. As a result, tailing is observed and the log_10_ reduction achieved at these fluences should be considered a minimum. The inactivation rate constants at 60 minutes post-exposure were statistically different for all UV sources (*p* < 0.05) except 255 nm vs. 265 nm (*p* = 0.13) and 265 nm vs. 285 nm (*p* = 0.68). However the *F-*test comparing 255 nm and 265 nm found the curves should not be fit with the same curve (*p* < 0.0001) and that the y-intercept was statistically different for each wavelength. The similarity of 265 nm and 285 nm was confirmed by the *F*-test (*p* = 0.76).

Log_10_ reduction continued to increase with time and after 180 minutes there is no longer a shoulder at low fluences at 265 nm and 285 nm, confirming that cercariae were damaged at 50 mJ/cm^2^. This suggests that cercariae are not able to repair UV induced damage in the dark, after having been exposed to these fluences. There was also an increase at 253.7 nm and 255 nm at 50 mJ/cm^2^, however it was minimal. As with the 60 minutes data, the inactivation rate constants at 180 minutes post exposure were statistically different for all UV sources (*p* < 0.05) except 255 nm vs. 265 nm (*p* = 0.85) and 265 nm vs. 285 nm (*p* = 0.23). The subsequent *F-*tests found 255 nm and 265 nm should not be fit with the same curve (*p* < 0.0001) but 265 nm and 285 nm were statistically similar (*p* = 0.26).

### Effect of stirring

There was a 2% difference between the proportion of live cercariae in samples stirred for 90 minutes when compared to unstirred samples immediately after stirring. This increased to 8% and 11% at 60 and 180 minutes after stirring, respectively, however the difference was only statistically significant at 180 minutes when compared with a *t-*test (*p* = 0.007). The difference between samples stirred for 10 and 30 minutes and unstirred controls was less than 1% and was not significant at any time point (*p* > 0.05).

## Discussion

These results show that very high fluences are required to kill *S*. *mansoni* cercariae at all the wavelengths studied. When studied immediately after exposure the 265 nm LEDs were the most effective, requiring a fluence of 247 mJ/cm^2^ to achieve a 1-log_10_ reduction. This level of inactivation was not achieved at any other wavelength at 0 minutes post exposure; however, the data suggests that cercariae were damaged as there was an increase in log_10_ reduction at 60 and 180 minutes post-exposure at all fluences and wavelengths. This agrees with a previous study by Ariyo and Oyerinde (1990) that found activity of *S*. *mansoni* cercariae significantly decreased 60 minutes after short exposure to UV light and death rates increased four hours after exposure [45].

After the 265 nm LEDs, the 285 nm LEDs were most effective, followed by the 255 nm LEDs and finally the LP lamp (253.7 nm). However, there was no statistical difference between the inactivation rate constants at 265 nm and 285 nm after 60 minutes, and slightly higher levels of inactivation were achieved at 285 nm, although all cercariae died in some samples so we can only determine the minimum log_10_-reduction achieved at 200 and 300 mJ/cm^2^ for these wavelengths. The fluence response of the LP lamp and 255 nm LEDs is visually similar up to a fluence of 150 mJ/cm^2^, but at high fluences 255 nm was more effective and the inactivation rate constants were statistically different, even though the peak wavelengths are very close.

Long periods of stirring (> 30 minutes) in non-UV exposed samples resulted in a statistically significant increase in dead cercariae, suggesting stirring may have contributed to the log_10_ reduction achieved in some UV exposed samples (e.g. samples exposed to 300 mJ/cm^2^ at 255 nm). This could explain why the fluence-responses at 253.7 nm and 255 nm diverge at 200 and 300 mJ/cm^2^. The average exposure times at 255 nm at these fluences were 62 minutes 50 seconds and 94 minutes 48 seconds respectively, compared to 16 minutes 31 seconds and 24 minutes 47 seconds at 253.7 nm. Cercariae are non-feeding, and they are shed from snail hosts with finite glycogen stores that are used for host finding and skin penetration [48]. They are stimulated by water turbulence [49], so it is possible that they utilize their glycogen stores more quickly in stirred samples and die earlier. Alternatively, the difference in efficacy could be due to the polychromatic output of the 255 nm LEDs compared to the monochromatic LP lamp. The emission spectrum of the 255 nm LEDs extends beyond 270 nm (Fig 1), with a considerable output at 260–265 nm, the range of maximum absorption of UV light by the pyrimidine bases.

The fluences required to achieve a 1- to 2-log_10_ reduction in cercariae in this study were much higher than required to achieve the same reduction in worm burden in previous vaccine studies (5–14 mJ/cm^2^ at 253.7 nm for a 1-log_10_ reduction, although fluences should be considered approximate [27–36]). Similar differences have been observed in other helminths such as *Ascaris suum*, where 100 mJ/cm^2^ was required to achieve a 1-log_10_ reduction in viable embryos but only 11 mJ/cm^2^ was required to achieve a 1-log_10_ reduction in worm burden [38,50]. The difference between these studies was attributed to a delay in the expression of UV-induced DNA damage during cell replication and incomplete recovery of worms from the animal host [38]. A scanning electron microscopy (SEM) study with *S*. *mansoni* found that worms developed from cercariae exposed to UV light at 253.7 nm for 2 minutes had abnormal features and damaged teguments [51], whilst a SEM study on cercariae exposed to the same wavelength for 2–3 minutes found that irradiated cercariae were morphologically the same as controls [52]. The UV fluences were not recorded in either study, but authors suggest the abnormalities in adult worms were a result of either UV-induced DNA damage causing mutagenic effects that do not appear until the adult stage or an enhanced immune response to irradiated cercariae by the host. However, schistosome cercariae do not undergo DNA replication or transcription until they transform into schistosomula upon skin penetration [53], so DNA damage is unlikely to be the cause of visible damage or death of cercariae.

Whilst cercariae do not undergo transcription, RNA is present, and it is assumed that transcripts are formed prior to release from snail hosts, allowing translation to continue [54]. For most organisms (excluding organisms in which RNA is the only genetic material, such as RNA viruses) UV damage to RNA is generally considered inconsequential relative to DNA because it exists in many copies and can be replaced so long as the DNA is unaffected [22]. However, in the case of cercariae, RNA cannot be replaced until transformation into schistosomula. A study by Hagerty *et al*. (2019) found that the majority of protein translation occurs in the tails of cercariae, possibly to maintain the energy required for swimming, and treatment with high concentrations of drugs that inhibit protein synthesis reduced activity [54]. RNA damage preventing protein translation could be a UV disinfection mechanism in schistosome cercariae and concentration of translation in the tail could possibly explain why head-tail detachment was observed in many samples 60 minutes after exposure to low fluences that did not kill cercariae heads. Whilst this is speculative, UV-C radiation has been shown to indirectly inhibit translation in mammalian cells [55–57] and previous studies investigating UV-attenuated cercarial vaccines found that non-lethal UV exposure at 253.7 nm prevented protein synthesis in schistosomula that had been transformed from irradiated cercariae [58]. Schistosome cercariae are relatively large (approximately 500 μm long, 20 μm diameter) and complex microorganisms, made up of over 1000 cells. Given their size it is possible the effects of UV exposure are concentrated in cells near the surface membrane [22]. UV light has been shown to cause aggregation of the glycocalyx and modify carbohydrates and glycoproteins on the surface of schistosomula transformed from irradiated cercariae [59–62].

It is likely that exposure to different wavelengths of UV light results in different or multiple disinfection mechanisms. A recent study with *Escherichia coli* found that exposure to UV LEDs with a peak emission at 265 nm caused direct damage to DNA, whereas UV LEDs with peaks at 285 nm and 295 nm caused indirect damage to cellular components through oxidative stress, possibly through the production of intracellular reactive oxygen species. Oxidative stress was also caused at 265 nm and direct DNA damage by the longer wavelengths, but the other mechanisms were dominant [26]. Medium pressure (MP) arc discharge lamps, which produce polychromatic UV light over a broad spectrum, have also been shown to cause oxidative stress in bacteria [25]. UV exposure can also cause damage to proteins, which strongly absorb UV at wavelengths less than 230 nm with a smaller peak in absorbance at approximately 280 nm [22]. Studies have shown that MP lamps and UV LEDs with peak emissions at 261 nm and 278 nm caused higher levels of protein damage in adenoviruses than LP lamps [23,24].

The sensitivity of a microorganism to UV light at different wavelengths is a function of the microorganism itself. We examined the effectiveness of UV disinfection against *S*. *mansoni* cercariae, which is one of the most widespread human schistosome species, present in South America, the Caribbean, the Middle East, and Africa. However further study of the other main human species, *S*. *haematobium* and *S*. *japonicum*, is required to determine if there is a difference in fluence response. Previous studies did not find any difference in the UV sensitivity of *S*. *mansoni* and *S*. *japonicum* cercariae [33]; *S*. *haematobium* has been shown to be slightly more resistant than *S*. *mansoni* [44], although no statistical analysis was performed. Whilst cercariae of the human schistosomes are physiologically similar, genomic differences could result in varying UV sensitivity between species, particularly if damage to nucleic acids is a principal disinfection mechanism.

The maximum log_10_ reduction achieved in this study was 2.1 representing > 99% removal. As all the cercariae were dead in these samples it is possible a higher level of inactivation could be reached. This was only achieved one to three hours post exposure for the UV LEDs, and not at all for the LP lamp. Viability was determined by assessing morphology and motility, this is a common method used to assess schistosome cercariae, but it can be subjective. Furthermore, assuming that all living cercariae are still infective even when they are visibly damaged is likely conservative. We are unable to confirm if cercariae that died at 60 or 180 minutes post-exposure were infective at 0 minutes post-exposure, but it has been demonstrated previously that older, non-swimming cercariae can still penetrate skin [48], so we consider this conservative approach to be appropriate from a public health protection standpoint. Future studies could use human skin samples obtained from cosmetic surgery to study skin penetration [63]. Fluorescence assays could also be used to assess the integrity of cell membranes following UV exposure to determine if damaged cercariae are non-viable [64] and immunological assays to detect nucleic acid damage are also worth investigating [65,66]. Both these assays have the additional benefit of being objective and could provide an insight into the possible UV disinfection mechanisms in schistosome cercariae at different wavelengths.

The concentration of cercariae in water bodies varies significantly and is dependent on many complex factors, but previous studies have reported ranges from < 0.1 to over 100 cercariae/L [67–69]. This is much lower than the average concentration of cercariae used in this study (29 cercariae/mL), therefore the fluence required to achieve a 2-log_10_ reduction may actually be sufficient to inactivate all cercariae in water that is used for contact activities (e.g. laundry, bathing). Furthermore, UV disinfection is normally preceded by some form of pretreatment, such as filtration, which reduces the turbidity and UV absorbance of the water and is likely to reduce the cercarial concentration even further. Nevertheless, the high fluences required to kill *S*. *mansoni* cercariae indicate that UV disinfection is unlikely to be an efficient method for providing water that is immediately free of schistosome cercariae, particularly compared to the relatively low contact times required to achieve similar levels of disinfection using chlorination [70]. Improvements in wall plug efficiencies combined with cheaper production costs may make UV LED technology more competitive, in terms of the whole life energy and cost requirements, in the near future [17–21].

## Conclusions

To the best of our knowledge this is the first reproducible study to test the direct effect of germicidal UV light on *S*. *mansoni* cercariae. We have demonstrated that very high fluences are required to kill *S*. *mansoni* cercariae even at the most effective wavelength, requiring 247 mJ/cm^2^ (95% CI: 234–261 mJ/cm^2^) to achieve a 1-log_10_ reduction at 265 nm. This reduces to 127 mJ/cm^2^ (95% CI: 111–146 mJ/cm^2^) and 99 mJ/cm^2^ (95% CI: 85–113 mJ/cm^2^) if cercariae are stored for one and three hours, respectively. 285 nm was found to be slightly less effective at 0 minutes post-exposure, but there was no statistical difference between 265 nm and 285 nm after 60 minutes. 255 nm and 253.7 nm were the least effective wavelengths. Whilst the concentration of cercariae used in this study is unlikely to occur in the environment, and total death of cercariae may be considered a conservative measure of UV effectiveness, we believe this is the correct approach from a public health standpoint.

Improvements in WASH infrastructure are required for sustainable control and elimination of schistosomiasis and other neglected tropical diseases, and the emerging technology of UV LEDs has the potential to provide chemical-free, point-of-use water treatment that is suitable for remote and off-grid locations. However, the high fluences required to kill *S*. *mansoni* cercariae suggest that UV disinfection is unlikely to be an energy- or cost-efficient water treatment method for preventing transmission of schistosomiasis on its own, unless there are improvements in UV LED technology or future studies demonstrate that cercariae damaged by UV are non-infective, research that is ongoing by the authors.

## Supporting information

S1 Fig UV fluence response of *S. mansoni* cercariae 0 minutes post exposure. Red symbols indicate data points that were not included in linear regression because they were outside the linear range or because all cercariae in the sample were dead. Equation represents log inactivation (y) as a function of fluence (x).
(TIFF)

S2 Fig UV fluence response of *S. mansoni* cercariae 60 minutes post exposure. Red symbols indicate data points that were not included in linear regression because they were outside the linear range or because all cercariae in the sample were dead. Equation represents log inactivation (y) as a function of fluence (x).
(TIFF)

S3 Fig UV fluence response of *S. mansoni* cercariae 180 minutes post exposure. Red symbols indicate data points that were not included in linear regression because they were outside the linear range or because all cercariae in the sample were dead. Equation represents log inactivation (y) as a function of fluence (x).
(TIFF)

S1 Table Inactivation rate constants were compared using a one-way ANOVA followed by Tukey’s multiple comparisons test. If the slopes of two lines were statistically similar (*p* > 0.05), an extra-sum-of-squares *F*-test was carried out to determine if the two data sets could be fit to a shared model, to confirm similarity.
(DOCX)
