# Supplementary figures and images for: Ultraviolet disinfection of Schistosoma mansoni cercariae in water
Source: PLoS Negl Trop Dis. 2021 Jul 6;15(7):e0009572. doi: 10.1371/journal.pntd.0009572 (PMC8284627; doi:10.1371/journal.pntd.0009572)

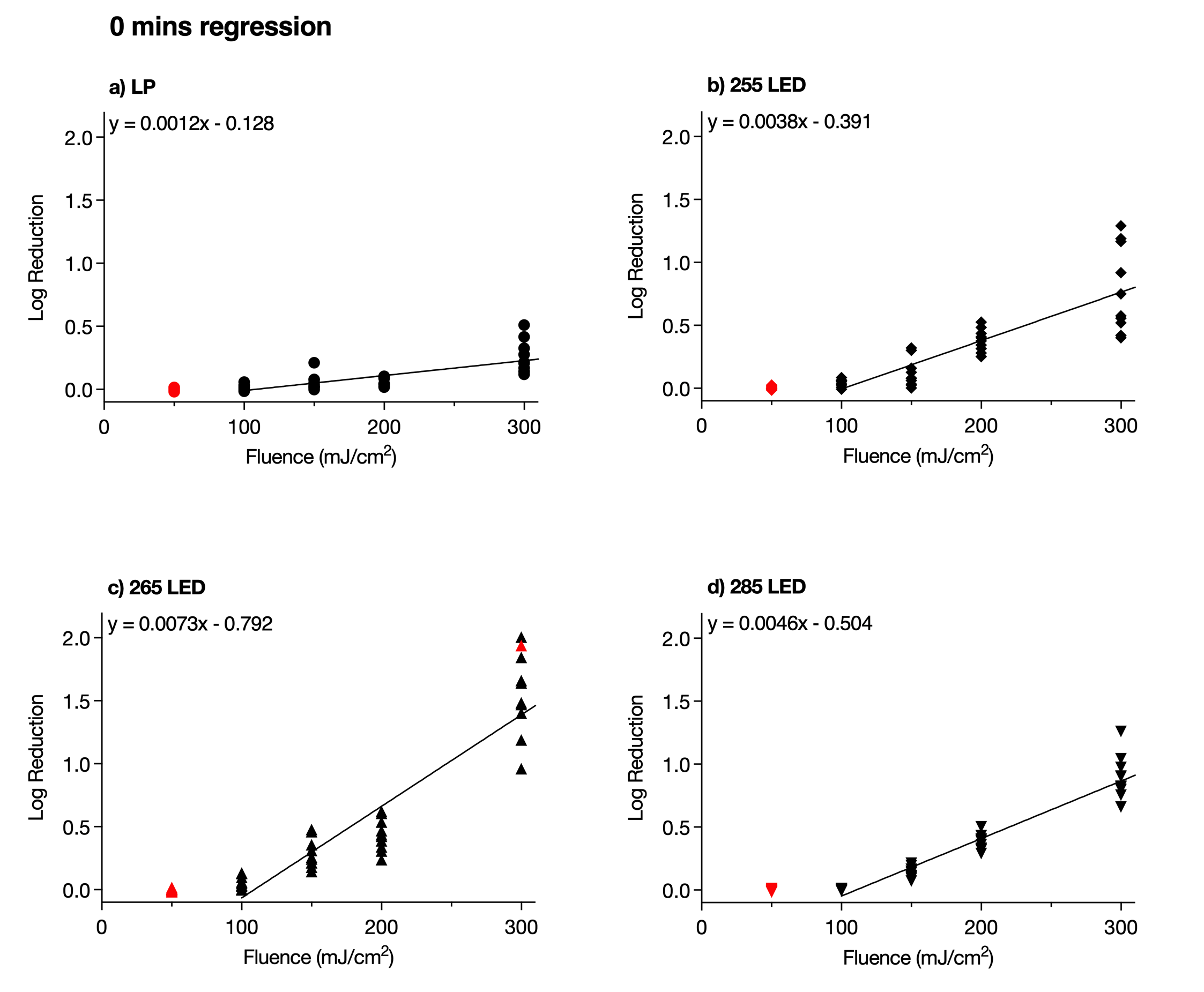

Supplement: S1 Fig — Red symbols indicate data points that were not included in linear regression because they were outside the linear range or because all cercariae in the sample were dead. Equation represents log inactivation (y) as a function of fluence (x). (TIFF) [file pntd.0009572.s001.tiff]

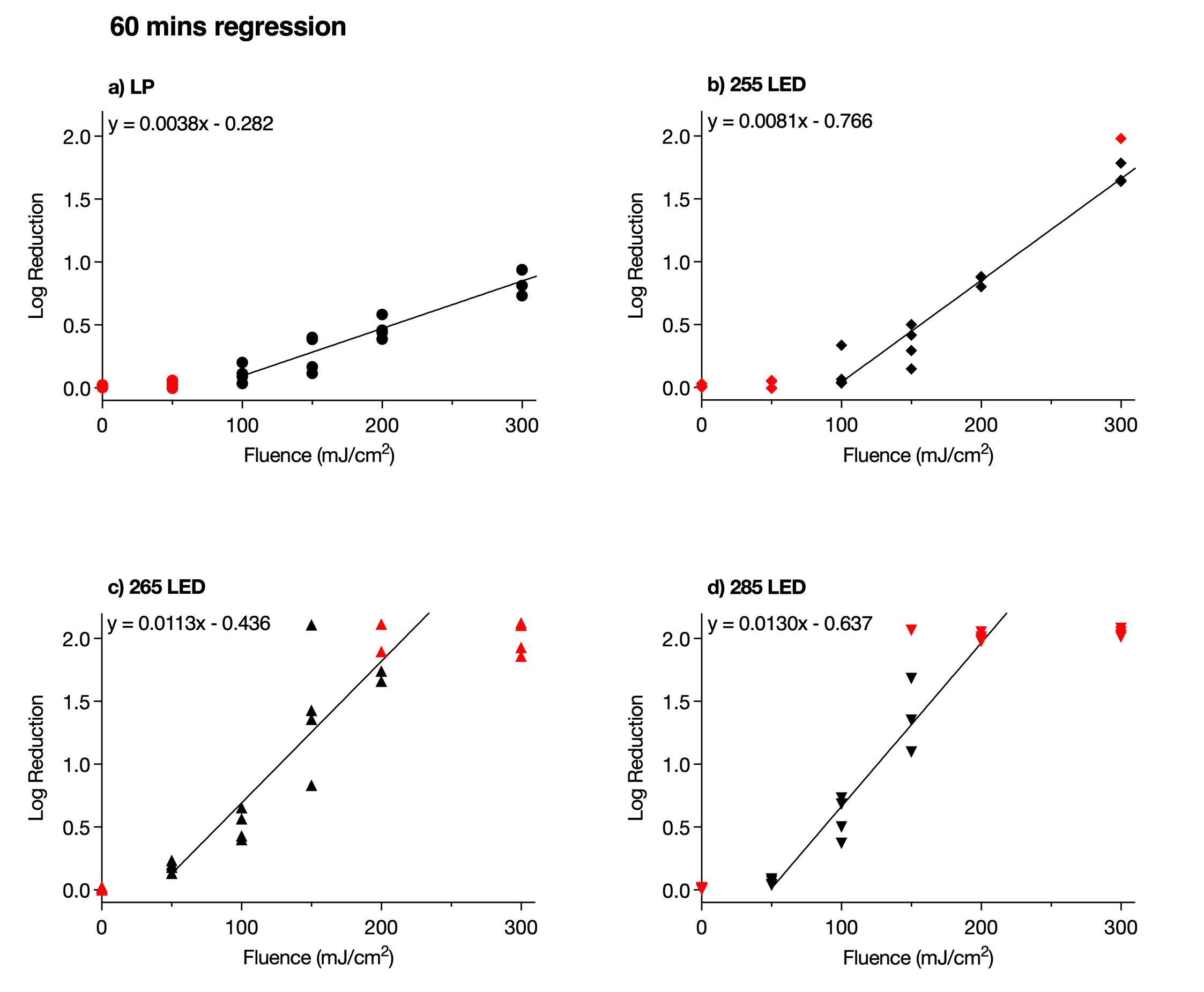

Supplement: S2 Fig — Red symbols indicate data points that were not included in linear regression because they were outside the linear range or because all cercariae in the sample were dead. Equation represents log inactivation (y) as a function of fluence (x). (TIFF) [file pntd.0009572.s002.tiff]

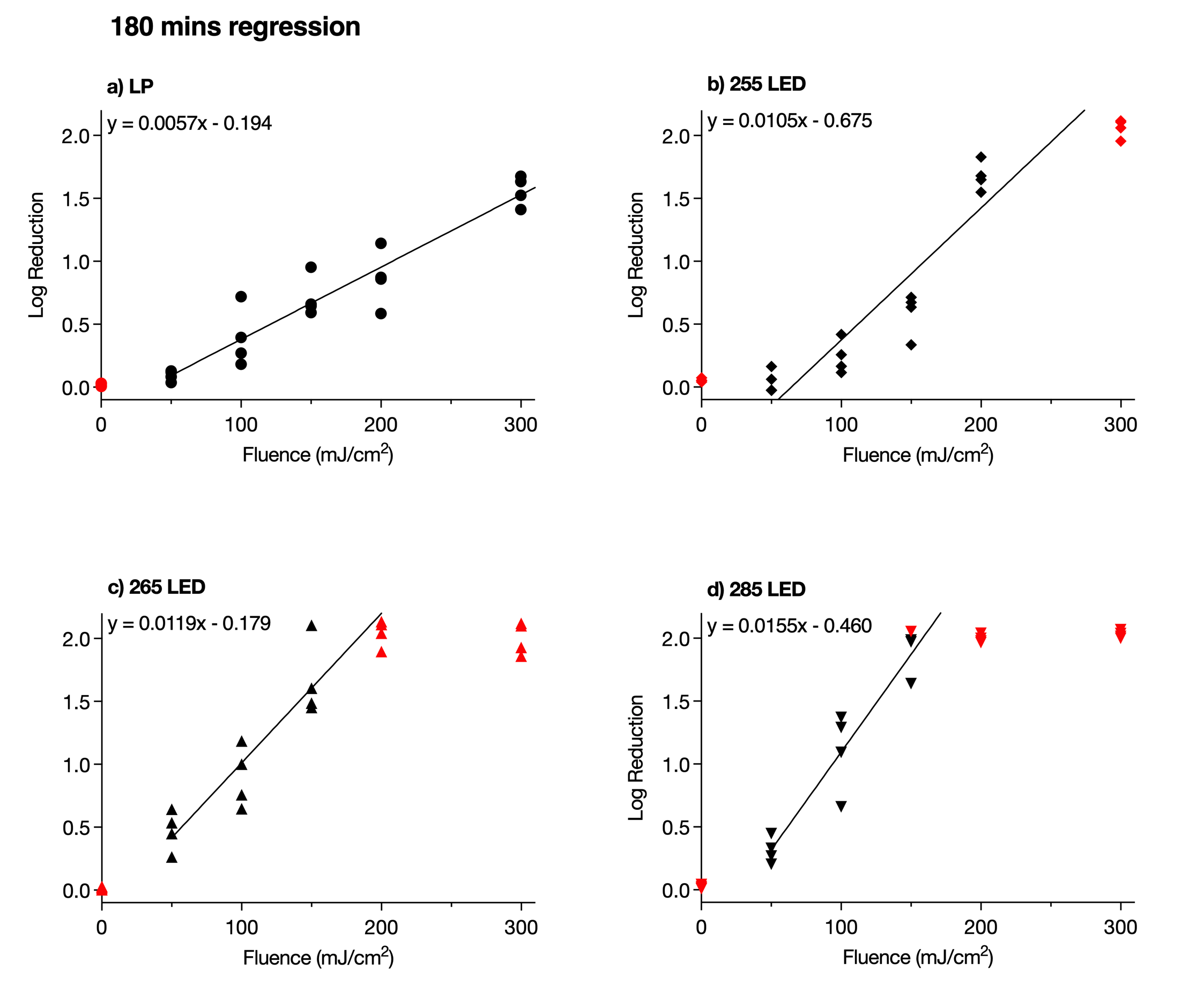

Supplement: S3 Fig — Red symbols indicate data points that were not included in linear regression because they were outside the linear range or because all cercariae in the sample were dead. Equation represents log inactivation (y) as a function of fluence (x). (TIFF) [file pntd.0009572.s003.tiff]
