# Supplementary material for: Ultraviolet disinfection of Schistosoma mansoni cercariae in water
Source: PLoS Negl Trop Dis. 2021 Jul 6;15(7):e0009572. doi: 10.1371/journal.pntd.0009572 (PMC8284627; doi:10.1371/journal.pntd.0009572)
Supplement: S1 Table — If the slopes of two lines were statistically similar (p > 0.05), an extra-sum-of-squares F-test was carried out to determine if the two data sets could be fit to a shared model, to confirm similarity. (DOCX) [file pntd.0009572.s004.docx]

**Ultraviolet disinfection of *Schistosoma mansoni* cercariae in water**

**Lucinda Hazell, Fiona Allan, Aidan M. Emery, and Michael R. Templeton**

**Supporting Information**

**S1 Table.** **Inactivation rate constants were compared using a one-way ANOVA followed by Tukey’s multiple comparisons test.** If the slopes of two lines were statistically similar (*p* > 0.05), an extra-sum-of-squares *F*-test was carried out to determine if the two data sets could be fit to a shared model, to confirm similarity.

| **Tukey's multiple comparisons test of inactivation rate constants (*k_D_*)** | | |
| --- | --- | --- |
| **0 mins** | **Adjusted P Value** | **Significance** |
| LP vs. 255 LED | <0.0001 | **** |
| LP vs. 265 LED | <0.0001 | **** |
| LP vs. 285 LED | <0.0001 | **** |
| 255 LED vs. 265 LED | <0.0001 | **** |
| 255 LED vs. 285 LED | 0.4500 | ns |
| 265 LED vs. 285 LED | <0.0001 | **** |
|  | | |
| **60 mins** | **Adjusted P Value** | **Significance** |
| LP vs. 255 LED | 0.0145 | * |
| LP vs. 265 LED | <0.0001 | **** |
| LP vs. 285 LED | <0.0001 | **** |
| 255 LED vs. 265 LED | 0.1277 | ns |
| 255 LED vs. 285 LED | 0.0119 | * |
| 265 LED vs. 285 LED | 0.6786 | ns |
|  | | |
| **180 mins** | **Adjusted P Value** | **Significance** |
| LP vs. 255 LED | 0.0128 | * |
| LP vs. 265 LED | 0.0024 | ** |
| LP vs. 285 LED | <0.0001 | **** |
| 255 LED vs. 265 LED | 0.8473 | ns |
| 255 LED vs. 285 LED | 0.0309 | * |
| 265 LED vs. 285 LED | 0.2309 | ns |
|  | | |
| **Extra sum-of-square *F-*test of slope (*k_D_*) and y-intercept (*c*)** | | |
| **0 mins** | **P Value** | **Significance** |
| 255 LED vs. 285 LED | 0.2316 | ns |
|  | | |
| **60 mins** | **P Value** | **Significance** |
| 255 LED vs. 265 LED | <0.0001 | **** |
| 265 LED vs. 285 LED | 0.7587 | ns |
|  | | |
| **180 mins** | **P Value** | **Significance** |
| 255 LED vs. 265 LED | <0.0001 | **** |
| 265 LED vs. 285 LED | 0.2598 | ns |
